# Supplementary material for: Sorting at embryonic boundaries requires high heterotypic interfacial tension
Source: Nat Commun. 2017 Jul 31;8:157. doi: 10.1038/s41467-017-00146-x (PMC5537356; doi:10.1038/s41467-017-00146-x)
Supplement: Supplementary file 2 — Supplementary Software 1 [file 41467_2017_146_MOESM2_ESM.zip › PottsModel/SrcPottsModel/doc/index-files/index-19.html]

T-Index


JavaScript is disabled on your browser.


Skip navigation links


- Overview
- Package
- Class
- Use
- Tree
- Deprecated
- Index
- Help

- Prev Letter
- Next Letter

- Frames
- No Frames

- All Classes

A B C D E F G H I J K L M N O P R S T U V W 


## T

targetArea - Variable in class model.Constants.ParameterManager


targetPerimeter - Variable in class model.Constants.ParameterManager


testGetAngleFromHorizontal() - Method in class model.VectorTests


testMeasureAngle() - Method in class model.VectorTests


toAxial() - Method in class gui.Hexagon.CubeCoordinates


toAxial() - Method in class gui.Hexagon.OffsetCoordinates


toCube() - Method in class gui.Hexagon.AxialCoordinates


toCube() - Method in class gui.Hexagon.OffsetCoordinates


toOffset() - Method in class gui.Hexagon.AxialCoordinates


toOffset() - Method in class gui.Hexagon.CubeCoordinates


toRGB(float[]) - Static method in class utils.HSLColor
:   Convert HSL values to a RGB Color with a default alpha value of 1.

toRGB(float[], float) - Static method in class utils.HSLColor
:   Convert HSL values to a RGB Color.

toRGB(float, float, float) - Static method in class utils.HSLColor
:   Convert HSL values to a RGB Color with a default alpha value of 1.

toRGB(float, float, float, float) - Static method in class utils.HSLColor
:   Convert HSL values to a RGB Color.

toString() - Method in class gui.Hexagon.OffsetCoordinates


toString() - Method in class gui.PixelDisplay


toString() - Method in class model.CartesianCoordinates


toString() - Method in class model.Cell


toString() - Method in class model.Constants


toString() - Method in interface model.Coordinates


toString() - Method in class model.FiniteVector


toString() - Method in class model.HalfLine


toString() - Method in class model.Lattice


toString() - Method in class model.Pixel


toString() - Method in class utils.HSLColor


TypeSpecificAreaStatistic - Class in engine


TypeSpecificAreaStatistic(PottsEngine, Cell.CellType[], int) - Constructor for class engine.TypeSpecificAreaStatistic


TypeSpecificCellStatistic - Class in engine


TypeSpecificCellStatistic(PottsEngine, String, Cell.CellType[], int) - Constructor for class engine.TypeSpecificCellStatistic


TypeSpecificNearestNeighborStatistic - Class in engine


TypeSpecificNearestNeighborStatistic(PottsEngine, Cell.CellType[], int, int) - Constructor for class engine.TypeSpecificNearestNeighborStatistic


TypeSpecificNearestNeighborStatistic(PottsEngine, Cell.CellType[], int) - Constructor for class engine.TypeSpecificNearestNeighborStatistic


TypeSpecificNumNeighborsStatistic - Class in engine


TypeSpecificNumNeighborsStatistic(PottsEngine, String, Cell.CellType[], int) - Constructor for class engine.TypeSpecificNumNeighborsStatistic


TypeSpecificPercentIsolatedCellStatistic - Class in engine


TypeSpecificPercentIsolatedCellStatistic(PottsEngine, String, Cell.CellType[], int) - Constructor for class engine.TypeSpecificPercentIsolatedCellStatistic


TypeSpecificPerimeterStatistic - Class in engine


TypeSpecificPerimeterStatistic(PottsEngine, Cell.CellType[], int) - Constructor for class engine.TypeSpecificPerimeterStatistic


TypeSpecificStatistic - Class in engine


TypeSpecificStatistic(PottsEngine, String, Cell.CellType[], int) - Constructor for class engine.TypeSpecificStatistic

A B C D E F G H I J K L M N O P R S T U V W

Skip navigation links


- Overview
- Package
- Class
- Use
- Tree
- Deprecated
- Index
- Help

- Prev Letter
- Next Letter

- Frames
- No Frames

- All Classes
